# Psychological distress and cancer worry in unaffected relatives undergoing cascade testing with multigene panel testing

**DOI:** 10.1038/s10038-026-01464-z

**Published:** 2026-03-02

**Authors:** Kaori Kimura, Takeshi Kuwata, Yumie Hiraoka, Manami Matsukawa, Chikako Tomozawa, Miki Aitani, Yu Komura, Izumi Miki, Masashi Wakabayashi, Yosuke Furui, Hiroki Kondo, Kazumasa Saigoh, Yasuko Yamamoto, Kiwamu Akagi, Hiroto Narimatsu, Megumu Yokono, Shinsuke Amano, Kaori Muto, Fuji Nagami, Kazuto Kato, Riu Yamashita, Issei Imoto, Shinji Kosugi, Makoto Hirata, Takayuki Yoshino, Yoshiaki Nakamura

**Affiliations:** 1 Department of Genetic Medicine and Services, National Cancer Center Hospital East, Kashiwa, Japan; 2 Department of Genetic Medicine and Services, National Cancer Center Hospital, Tokyo, Japan; 3 Translational Research Support Office, National Cancer Center Hospital East, Kashiwa, Japan; 4 Biostatistics Division, Center for Research Administration and Support, National Cancer Center Hospital East, Kashiwa, Japan; 5 FALCO Biosystems, Kyoto, Japan; 6 Department of Clinical Genetics, Kinki University Hospital, Osaka, Japan; 7 Department of Hereditary Tumors, National Hospital Organization Shikoku Cancer Center, Matsuyama, Japan; 8 Center for Genomic Diagnosis, International University of Health and Welfare Narita Hospital, Narita, Chiba Japan; 9 Department of Genetic Medicine, Kanagawa Cancer Center, Yokohama, Kanagawa Japan; 10 School of Social Sciences, Waseda University, Tokyo, Japan; 11 Japan Federation of Cancer Patient Organizations, Yokohama, Japan; 12 Department of Public Policy, The Institute of Medical Science, The University of Tokyo, Tokyo, Japan; 13 Tohoku Medical Megabank Organization, Tohoku University, Sendai, Japan; 14 Department of Biomedical Ethics and Public Policy, Graduate School of Medicine, The University of Osaka, Osaka, Japan; 15 Division of Translational Informatics, Exploratory Oncology Research and Clinical Trial Center, National Cancer Center, Kashiwa, Japan; 16 Aichi Cancer Center Research Institute, Nagoya, Japan; 17 Department of Genomic Medicine, Kyoto University School of Public Health, Kyoto, Japan; 18 Department of Gastroenterology and Gastrointestinal Oncology, National Cancer Center Hospital East, Kashiwa, Japan; 19 Division for the Promotion of Drug and Diagnostic Development, National Cancer Center Hospital East, Kashiwa, Japan

**Keywords:** Genetic counselling, Genetic testing, Cancer genetics, Psychology

## Abstract

Multigene panel testing (MGPT) is increasingly used for diagnosing hereditary cancers; however, its application in cascade testing remains limited, and the psychological impact on unaffected relatives is not well understood. This multicenter study in Japan recruited 123 first-degree relatives without a personal history of cancer to evaluate psychological responses before (T0) and two weeks after (T1) MGPT result disclosure. Participants completed the Japanese version of the Cancer Worry Scale (CWS-J) at both time points and the Impact of Event Scale–Revised (IES-R) at T1. Two weeks after disclosure, 6.5% of relatives met the threshold for clinically significant distress. Mean IES-R scores were higher among carriers of pathogenic variants (mean 8.2) than among those with negative results (mean 4.8) or variants of uncertain significance (mean 2.4). In contrast, multivariate analysis revealed no independent association between test result and post-disclosure distress. In contrast, higher baseline CWS-J scores were strongly associated with elevated IES-R scores after disclosure (*p* < 0.001). Children and relatives who frequently discussed cancer risk within their families exhibited higher distress levels than other subgroups. Overall, CWS-J scores showed no significant change from pre- to post-disclosure, with 41% of relatives maintaining persistently high worry. Across both time points, unaffected relatives consistently reported lower CWS-J scores than individuals with cancer (*p* < 0.0001). These findings suggest that MGPT has minimal psychological impact on unaffected relatives undergoing cascade testing, although those with higher preexisting cancer worry may require additional psychosocial support.

## Introduction

As multigene panel testing (MGPT) is increasingly incorporated into clinical oncology [1], a clear understanding of its psychological impact on test recipients is essential. Prior studies indicate that relatives generally report lower levels of distress than probands [2], that cancer worry decrease among unaffected relatives following cascade testing [3], and that distress often rises immediately after result disclosure before decline over time [4]. Such psychological responses can influence screening behaviors [5] and quality of life [6]. The psychological effects of genetic testing are commonly measured using instruments such as the Cancer Worry Scale (CWS) [3, 7, 8], the Impact of Event Scale (IES) [2, 4], and the Multidimensional Impact of Cancer Risk Assessment (MICRA) [9–11].

Although clinical uptake of MGPT varies by country [12–15], its use in affected probands is widespread, and in recent years, its application to cascade testing for relatives has been increasingly explored [16, 17]. Existing research on relatives has largely focused on those identified as carriers of germline pathogenic variants (GPVs) [2] or on single-site testing approaches [2, 3]. Moreover, most MGPT-related psychological research has targeted affected probands [7, 10, 11], leaving the psychological impact of cascade testing using MGPT on unaffected first-degree relatives insufficiently characterized.

This study aims to clarify the psychological impact of cascade testing using MGPT on unaffected relatives of individuals with hereditary cancer. Specifically, it seeks to (1) assess the extent of genetic test–related distress following cascade testing using MGPT and examine its associations with demographic and clinical factors; (2) evaluate changes in cancer worry before and after the disclosure of test results; and (3) compare cancer worry between unaffected relatives and individuals with cancer at both pre- and post-disclosure time points.

## Methods

### Study design and participants

This psychological sub-study was conducted as part of the BRANCH study (UMIN000046085), which aimed to evaluate the clinical utility of MGPT in Japanese oncology practice among individuals with cancer and their first-degree relatives diagnosed with hereditary cancer.

Between June 2022 and October 2023, 49 institutions participated in the BRANCH study. As of October 2023, the BRANCH study comprised four cohorts (Supplementary Fig. 1). The eligibility criteria for participants included being ≥20 years of age between June and August 2022 and ≥18 years of age from September 2022 onward. The participants were required to be proficient in reading, writing, and speaking Japanese. The study protocol was approved by the Institutional Review Board of the National Cancer Center (2021-194) and the ethics committees of all participating institutions.

In this sub-study, we defined unaffected relatives as cancer-unaffected first-degree relatives of cancer-affected probands in whom GPVs were identified in pre-specified genes (Supplementary Table 2a). These unaffected relatives were derived from Cohort C of the BRANCH study. To assess the differences in cancer worry according to cancer history, individuals with cancer were defined as the control group. Individuals with cancer were derived from a subset of Cohort C and from Cohort D. In Japan, the majority of genetic tests for hereditary cancer are not covered by public health insurance. However, *BRCA1* and *BRCA2* testing (*BRCA1/2* testing) (Myriad Genetics, Salt Lake City, UT, USA) is covered by public insurance for individuals who meet specific clinical criteria. Individuals with cancer who fulfilled at least one of these criteria were included in the study. (1) individuals with cancer who had previously undergone *BRCA1/2* testing and received the results (Cohort D), or (2) first-degree relatives of patients with hereditary cancer with a personal history of cancer (a subset of Cohort C) (Supplementary Fig. 2).

### Study procedures

The study flow is presented in Supplementary Fig. 1. During the informed consent process, participants were given the option to receive either information limited to the proband’s variant or the full results of MGPT. Electronic informed consent was obtained from all participants, after which peripheral blood samples were collected. MGPT targeted 35 genes associated with hereditary cancer syndromes (Supplementary Table 2b). This MGPT includes hereditary tumor predisposition genes associated with increased risks of breast, ovarian, colorectal, and pancreatic cancer. Testing was conducted by FALCO Biosystems (Kyoto, Japan) and included the detection of single-nucleotide variants, insertions/deletions, and copy number variations. For unaffected relatives, single-site analysis was performed using Sanger sequencing or multiplex ligation-dependent probe amplification (MLPA) to identify known proband-specific pathogenic variants. Genetic testing results were classified according to the American College of Medical Genetics and Genomics guidelines [18], as follows:

(i) Pathogenic or likely pathogenic variant (GPV)

(ii) Variant of uncertain significance (VUS)

(iii) Negative (benign or likely benign)

Ten days after blood collection, participants completed the first electronic patient-reported outcome (ePRO) questionnaire (T0). At T0, participants responded to items assessing their demographic characteristics (marital status, presence of children, education level, and income), clinical information (personal and family history of cancer and current screening status) and Japanese version of the Cancer Worry Scale (CWS-J) (Supplementary Table 1).

The genetic testing results were returned to each institution. Based on each participant’s preference for the scope of disclosure, results were disclosed by the attending physician. Those who opted for MGPT disclosure received the results for all 35 genes, including VUS.

Two weeks after disclosure, participants completed the second ePRO (T1). At T1, they responded to the CWS-J, the revised Impact of Event Scale (IES-R), and additional questions regarding family discussions about cancer risk and satisfaction with the genetic testing experience (Supplementary Table 1).

Only participants who completed both T0 and T1 assessments were included in the analysis. Demographic and clinical data (sex, age, ethnicity, recurrence, metastasis) were collected using an electronic data capture system (Supplementary Table 1). Genetic test results were provided by FALCO Biosystems. Participants were categorized into three groups: GPV group (with or without coexisting VUS), VUS group (VUS only), and negative group (neither GPV nor VUS).

### Psychological assessment tools

The CWS is an eight-item questionnaire that assesses anxiety regarding cancer risk and its impact on daily life. Items were rated on a 4-point scale, and scores ≥14 were defined as high cancer worry (CWS-high) [19] (Supplementary Table 3). In this study, scores ≥14 and <14 were classified as CWS-high and CWS-low, respectively. CWS-J has demonstrated strong internal consistency (Cronbach’s α = 0.89) [20] and has been used in various high-risk cancer populations [7, 8, 21].

The IES-R is a 22-item scale that measures intrusion, avoidance, and hyperarousal symptoms related to post-traumatic stress disorder (PTSD) (fourth edition of the Diagnostic and Statistical Manual of Mental Disorders) [22]. Items were rated on a 4-point scale, yielding a total score of 0–75. Scores ≥25 indicate partial PTSD or clinically significant symptoms (Supplementary Table 3). In this study, an “event” was defined as the disclosure of genetic test results in the BRANCH study. IES-high was defined as a score ≥25 and IES-low as <25. The Japanese version demonstrated high internal consistency (Cronbach’s α = 0.95) [23] and has been widely used in populations that underwent cancer or genetic testing [2, 24, 25].

As most participants with cancer were presumed to have undergone *BRCA1/2* testing before enrollment, the IES-R was analyzed only in the unaffected relatives.

### Statistical analysis

Descriptive statistics were used to summarize demographic, clinical, and genetic data. For univariate analyses, Student’s *t* test was applied for two-arm comparisons, and analysis of variance (ANOVA) or Kruskal–Wallis tests were used for three or more group comparisons to examine associations between psychological measures and the test results. To adjust for confounding factors, multivariate logistic regression and multivariate linear regression models were performed by incorporating genetic test results and clinical factors from the BRANCH study. Statistical significance was set at *p* < 0.05. All the analyses were performed using SAS (version 9.4; Cary, NC, USA).

## Results

### Study participants

During the study period, 198 unaffected relatives were enrolled in the BRANCH study. Among them, 123 (62%) completed both the pre- and post-disclosure questionnaires. The control group comprised 128 individuals with cancer, of whom 67 (52%) completed the questionnaires. Compared with individuals with cancer, unaffected relatives were significantly more likely to be male, younger, and possess a university-level education (*p* < 0.01; Table 1).

**Table 1:** Respondent characteristics

	Unaffected relatives	Individuals with cancer	*p*-value
	*n* = 123 (%)	*n* = 67 (%)	
Age			
Median (range)	44 (18–76)	50 (20–77)	0.0011
Gender			
Male	42 (34.2)	2 (3.0)	<0.0001
Female	81 (65.9)	65 (97.0)	
Cancer of proband^a^			
Breast	39 (31.7)	–	
Pancreas	27 (22.0)	–	
Ovary	25 (20.3)	–	
Prostate	4 (3.3)	–	
Others	28 (22.8)	–	
Cancer status^b^			
Breast	–	54 (80.6)	
Pancreas	–	4 (6.0)	
Ovary	–	9 (13.4)	
Genetic testing results provided by BRANCH study			
Negative	44 (35.8)	23 (34.3)	0.9802
GPV	54 (43.9)	30 (44.8)	
VUS	25 (20.3)	14 (20.9)	
Marital status (partner)			
No	48 (39.0)	16 (23.9)	0.0752
Yes	74 (60.2)	51 (76.1)	
No answer	1 (0.8)	0 (0)	
Children			
No	75 (61.0)	48 (71.6)	0.1415
Yes	48 (39.0)	19 (28.4)	
Education level			
～Junior colleges	59 (48.0)	48 (71.6)	0.0051
University～	63 (51.2)	18 (26.9)	
No answer	1 (0.8)	1 (1.5)	
Frequency of cancer risk discussion with family members			
A lot	24 (19.5)	13 (19.4)	0.6135
Somewhat	68 (55.3)	39 (58.2)	
A little	24 (19.5)	9 (13.4)	
Not at all	6 (4.9)	6 (9.0)	
Don't remember	1 (0.8)	0 (0)	
Previous genetic testing			
No	123 (100)	2 (3.0)	<0.0001
Yes	0 (0)	65 (97.0)	
Previous *BRCA1,BRCA2* testing results^c^			
Negative	–	40 (59.7)	
GPV	–	25 (37.3)	
Relationship with proband^a^			
Father	4 (3.3)	–	
Mother	5 (4.1)	–	
Children	69 (56.1)	–	
Sibling /brother	45 (36.6)	–	

*Unaffected relatives* cancer-unaffected first-degree relatives of individuals with hereditary cancer, *GPV* Germline pathogenic variant, *VUS* Variant of uncertain significance

^a^Data were collected exclusively from unaffected relatives

^b^Data were collected exclusively from individuals with cancer

^c^*n* = 65 (previous genetic testing was “Yes”)

Among the unaffected relatives, non-carriers of GPVs (*p* < 0.05) and younger participants (*p* < 0.05) were significantly less likely to complete the questionnaires. No significant differences in response rates were observed based on gender, relationship with the proband, or receipt of genetic counseling (Supplementary Table 4).

The median age of the unaffected relatives was 44 years (range: 18–76 years), with 65.9% female and 56.1% identifying as children of the proband (Table 1). The most common cancer type of proband was breast cancer (31.7%), followed by pancreatic cancer (22.0%) and ovarian cancer (20.3%). GPVs were identified in 54 individuals (43.9%), with 85% of the cases involving *BRCA1* or *BRCA2*. Only one participant had a GPV that was not found in the proband. Approximately 75% of participants reported discussing cancer risk with family members “a lot” or “somewhat.” All participants opted to receive both single-site and MGPT results, and 85.4% reported being “satisfied” or “somewhat satisfied” with undergoing MGPT. The clinical and genetic characteristics of the 123 unaffected relatives and 67 individuals with cancer included in the analysis are summarized in Supplementary Table 5.

### Genetic testing-related distress in unaffected relatives

In the unaffected relatives group, 6.5% (8/123) of the participants met the IES-high threshold. All individuals were categorized as CWS-high in the post-disclosure and were children of the proband. Within GPV group, 11.1% (6/54) were IES-high compared with 4.6% in the negative group, and 0% in the VUS group.

**Fig. 1:**
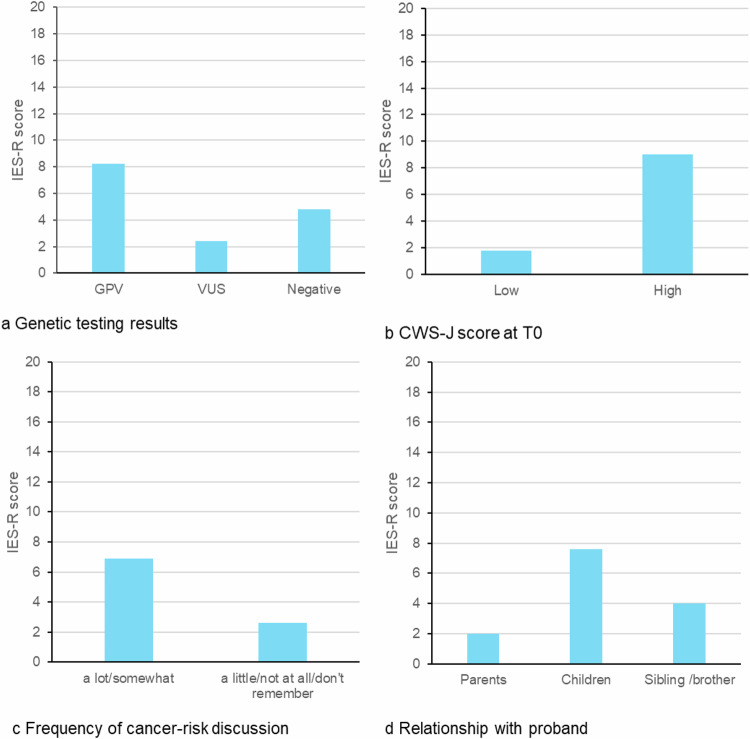
Genetic testing-related distress (IES-R) according to the background characteristics of the participants. IES-R scores were presented as background factors among cancer-unaffected first-degree relatives of individuals with hereditary cancer. IES-R scores were collected two weeks after the disclosure of test results. All results are shown as mean. **a** Genetic test result categories in the BRANCH study (*p* = 0.1386). (GPV, germline pathogenic variant; VUS, variant of uncertain significance). **b** Pre-disclosure (T0) cancer worry levels assessed using the Japanese version of the Cancer Worry Scale (CWS-J) and categorized as low (<13) and high (≥14) (*p* < 0.001). **c** Frequency of discussions with family regarding cancer risk (*p* < 0.05, univariate analysis). **d** Relationship with the proband (*p* = 0.0552)

Mean IES-R scores were highest in GPV group (Mean ± SD: 8.2 ± 11.6, range: 0–55), followed by the negative (4.8 ± 6.4, range: 0–26) and VUS groups (2.4 ± 5.4, range: 0–24). However, multivariate analysis revealed no significant association between genetic test results and IES-R scores (*p* = 0.1386) (Fig. 1, Supplementary Table 6).

Participants classified as CWS-high in the pre-test had significantly higher IES-R post-disclosure scores than those classified as CWS-low (9.0 ± 11.0 vs. 1.8 ± 2.7). Multivariate analysis confirmed a significant association between baseline cancer worry and post-disclosure distress (*p* < 0.001) (Fig. 1, Supplementary Table 6).

Participants who frequently discussed cancer risk with their families had significantly higher IES-R scores than those who did not (6.9 ± 9.9 vs. 2.6 ± 5.5, *p* < 0.05) (Fig. 1, Supplementary Table 6). Children of the proband showed higher IES-R scores (7.6 ± 11.1) than parents (2.0 ± 1.9) and siblings (4.0 ± 5.4) (Fig. 1). Univariate analysis indicated a trend toward higher IES-R scores among the children of the proband (*p* = 0.0517), which was similarly observed in the multivariate analysis (*p* = 0.0552) (Supplementary Table 6). Although the difference was not statistically significant, women had higher IES-R scores (6.1 ± 9.2) than men (5.3 ± 9.2) and participants whose probands were diagnosed with pancreatic cancer had higher mean IES-R scores (8.7 ± 10.1) than those with breast (5.2 ± 10.3) or ovarian cancer (5.5 ± 10.1) (Supplementary Table 6). A similar tendency was observed for the proband’s disease stage and genotype. Participants whose proband had stage IV cancer had slightly higher IES-R scores (6.3 ± 7.0) than those whose proband had stage III or earlier disease (5.9 ± 10.5) (*p* = 0.5836). Participants whose proband carried a GPV in *BRCA1* or *BRCA2* had lower IES-R scores (5.5 ± 9.5) than those whose proband carried GPVs in other genes (7.3 ± 7.7) (*p* = 0.2308) (data not shown).

### Changes in cancer worry among unaffected relatives

**Fig. 2:**
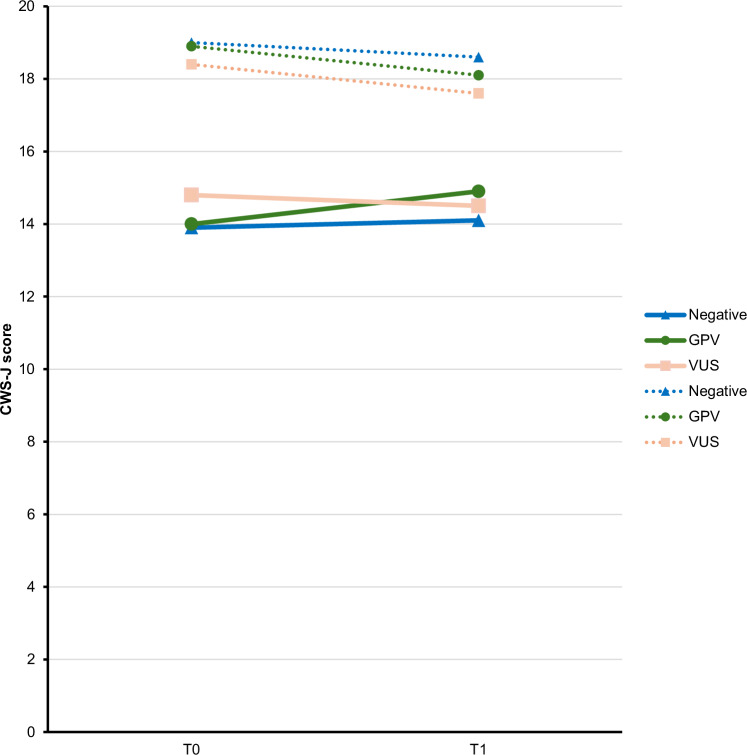
Changes in cancer worry before and after the disclosure of genetic testing results provided by the BRANCH study. The solid line indicates cancer-unaffected first-degree relatives of individuals with hereditary cancer, and the dashed line indicates individuals with cancer. T0 pre-disclosure of genetic testing (baseline), T1 two weeks after result disclosure, GPV Germline pathogenic variant, VUS Variant of uncertain significance, CWS-J Japanese version of the Cancer Worry Scale

In the group of unaffected relatives, 41% (51/123) reported persistently high cancer worry (CWS-high) in both the pre- and post-result disclosure assessments. Of these unaffected relatives, 35% (18/51) tested negative in the BRANCH study. The proportion of CWS-high was higher in women (44.4%, 36/81) than in men (35.7%, 15/42). Additionally, 49.6% (61/123) reported high cancer worry after disclosure but had low distress levels. Participants who frequently discussed cancer risk with their families had a higher proportion of IES-low and CWS-high scores (58.7%, 54/92) than those who did not (22.6%, 7/31). Participants whose proband had pancreatic cancer were more likely to show CWS-high (51.9%, 14/27) than those whose proband had breast cancer (30.8%, 12/39). Furthermore, participants whose probands had stage IV cancer were more likely to show CWS-high (61.0%, 25/41) than those whose proband had stage III or earlier disease (43.2%, 32/74). Among participants whose probands carried a GPV in *BRCA1* or *BRCA2*, the proportion of CWS-high was 46.4% (45/97), whereas it was 61.5% (16/26) among those whose probands carried GPVs in other genes. All participants had IES-low (Supplementary Table 7). The mean change in the CWS-J score was the highest in GPV group (T1–T0: +0.9), followed by the negative group (+0.2) and the VUS group (–0.3) (Fig. 2, Supplementary Table 8), but none of these differences were statistically significant (*p* = 0.9915).

### Comparison of cancer worry between unaffected relatives and individuals with cancer

**Fig. 3:**
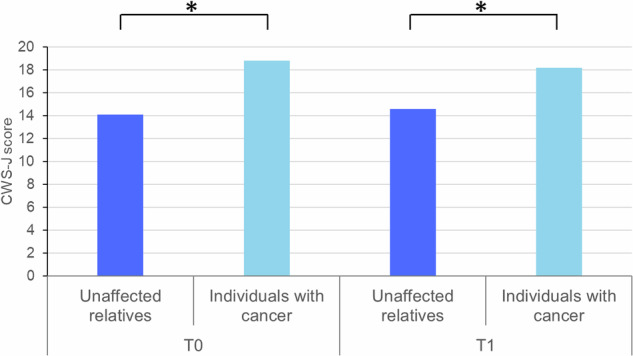
Cancer worry scores before and after genetic testing result disclosure in unaffected relatives and individuals with cancer. All results are shown as mean. T0, pre-disclosure of genetic testing (baseline); T1, two weeks after result disclosure; CWS-J, Japanese version of the Cancer Worry Scale. unaffected relatives, cancer-unaffected first-degree relatives of individuals with hereditary cancer. unaffected relatives (*N* = 123). individuals with cancer (*N* = 67). *p < 0.0001

The CWS-J scores were significantly higher in individuals with cancer than in unaffected relatives at both time points (pre- and post-result disclosure). At pre-disclosure, the mean CWS-J score was 14.1 ± 4.0 for unaffected relatives and 18.8 ± 5.5 for individuals with cancer; after disclosure, the scores were 14.6 ± 4.7 and 18.2 ± 5.1, respectively (*p* < 0.0001) (Fig. 3).

In contrast, the change in the CWS-J score from pre- to post-disclosure was not significantly associated with cancer history (unaffected relatives: +0.4 ± 4.2; individuals with cancer: –0.6 ± 4.0; *p* = 0.3105).

## Discussion

This study demonstrates that MGPT used in cascade testing has minimal overall psychological impact on unaffected relatives of individuals with hereditary cancer and supports its consideration as an alternative to single-site testing in cascade programs. To our knowledge, this is the first prospective study to evaluate the psychological impact of cascade testing using MGPT in unaffected relatives. While MGPT is traditionally applied for diagnostic testing in affected probands, its use in cascade testing can identify GPVs or VUS not detected in the proband [16, 17]. Despite this potential, the psychological consequences of applying MGPT in cascade settings for unaffected relatives have not been well characterized. In the United States, MGPT has increasingly been implemented clinically beyond single-site testing for proband-identified GPVs, including testing of unaffected individuals [15], whereas in Japan, public insurance currently covers only single-gene testing for a subset of individuals with cancer. Traditionally, cascade testing for hereditary cancer has relied on single-site assays; however, MGPT enables more comprehensive screening of inherited risk from both maternal and paternal lineages and may thus represent a viable alternative. We also observed that baseline cancer worry among unaffected relatives was associated with post-disclosure distress, and that cancer worry was lower in unaffected relatives than in individuals with cancer. Prior studies of the psychological impact of MGPT have largely focused on affected individuals [7, 10, 11], our findings contribute to the limited evidence regarding the psychological effects of MGPT in unaffected relatives [4]. However, the findings should be interpreted with caution. The unaffected participants in this study were first-degree relatives of individuals with hereditary cancers, and the possibility of carrying additional GPVs other than the known one was very low. Consequently, the psychological responses observed in this cohort were likely to primarily reflect reactions to single-site cascade testing, and we might not be able to fully assess the psychological impact of the broader information provided by the MGPT.

Overall psychological distress among unaffected relatives was low, with only 6.5% classified as IES-high. This low level of distress may be attributable to several factors: most participants were aware of their 50% carrier probability, all participants opted to receive the complete MGPT results and may have recognized the benefits of obtaining more comprehensive genetic information for their health management, and 98% received genetic counseling, which likely reduced distress and improved comprehension [26, 27]. Only one relative harbored a GPV that differed from the proband. Thus, the scarcity of unexpected findings may partly explain the observed low distress. Nevertheless, a subset of GPV carriers experienced increased distress after result disclosure, indicating the need for individualized support. VUS carriers reported lower distress than those with negative results. Our findings are inconsistent with those of previous studies [8, 28]. The provision of explanatory materials and follow-up systems may have contributed to this difference, and the higher tolerance for ambiguity observed in Asian populations may have contributed to the mitigation of this distress [29].

Pre-result disclosure cancer worry was significantly associated with post-disclosure distress. This finding is consistent with those of previous studies in individuals with cancer [7]. This suggests that psychological responses are influenced by preexisting cancer worry, highlighting the importance of continuous psychological assessment from the pre-test phase to inform individualized support strategies.

The children of the proband had higher levels of distress than their siblings or parents, and all IES-high cases were among the children of the proband. Our results suggest that familial relationships are important factors in evaluating post-disclosure distress. While genetic testing uptake varies according to the relationship with the proband [30], differences in psychological responses have not been well-documented. This may be attributable to the fact that children are younger and more likely to harbor concerns about future reproductive planning and life events and may have been more directly affected by familial cancer experiences than other family members [31, 32]. Accordingly, psychological support should be tailored to the age of the individual and their familial relationship with the proband.

Participants whose proband had pancreatic cancer tended to report higher cancer worry at the post‑disclosure, although they did not meet the IES‑high threshold. A similar tendency toward higher cancer worry post‑disclosure was also observed among participants whose proband had advanced‑stage cancer. Pancreatic cancer is often difficult to detect early and typically has a poor prognosis; stage IV disease is likewise characterized by poor treatment response and proximity to death, and these clinical features uniformly indicate a common poor prognosis. Experiencing such prognostically unfavorable situations within the family might have contributed to elevated cancer worry after testing among unaffected relatives. While previous studies have focused on breast and ovarian cancers [3] and individuals with cancer [10, 33], this is the first study to compare the psychological impact of the proband’s cancer type, including pancreatic cancer, on unaffected relatives.

Regarding family communication, frequent discussions about cancer risk were associated with higher distress levels in some cases. Our findings are inconsistent with those of previous studies [34, 35]. In Asian cultural contexts, hereditary diseases are stigmatized [36–38]. Our findings suggest that cultural background, coupled with heightened emotional sensitivity or negative expressions during family conversations, may trigger distress. These findings underscore the importance of follow-up through genetic counseling to address emotional concerns and facilitate informed decision-making. Conversely, more than half of those who engaged in frequent family discussions exhibited low levels of distress but high levels of cancer worry. Engaging in detailed conversations may have led to a more specific perception of personal and familial cancer risk, thereby heightening cancer worry. Individuals with higher cancer worry and more frequent family communication are more likely to undergo cancer screening [5, 39], suggesting that the relationship among family communication, distress, and cancer worry is complex and requires further investigation.

Women showed a higher proportion of CWS-high both before and after result disclosure and had higher post-disclosure IES-R scores than men. This tendency might reflect that the GPVs identified in the probands, especially in *BRCA1*, *BRCA2*, and *ATM*, are associated with elevated risk of breast or ovarian cancer, which has a greater impact on women than men. Similarly, previous study reported higher levels of distress among women undergoing hereditary cancer testing [8].

Relatives of probands with non-*BRCA1/2* GPVs showed higher post-disclosure IES-R scores and a higher proportion of CWS-high compared with those with *BRCA1/2* GPVs. Non-*BRCA1/2* GPVs in this cohort included *ATM* and *BRIP1*, which are moderate penetrance genes. Previous studies have reported that, among patients themselves, moderate penetrance genes may be associated with a higher psychological burden than high penetrance genes [7]. Taken together, these findings suggest that even among unaffected relatives, ambiguity in clinical guidance regarding the risk assessment and management of moderate penetrance genes may influence post-disclosure IES-R and CWS-J scores. However, the number of relatives with non-*BRCA1/2* GPVs in our study was limited, and further research with a larger sample size is warranted.

Cancer worry did not change significantly from pre- to post-disclosure, which is consistent with previous reports on individuals with cancer [7]. Padmanabhan et al. reported a reduction in cancer worry among unaffected relatives following cascade testing for *BRCA1/2*; however, attrition in the post-test sample may have introduced bias [3]. In our study, 41% of participants exhibited high levels of cancer worry both before and after testing. This may be related to the fact that MGPT provides more information prior to testing than single-site analysis and that it may reveal pathogenic variants in genes different from those identified in the proband [40]. Many participants continued to experience elevated worry even after receiving a negative result, suggesting that factors independent of test outcomes, such as family history of cancer or feeling of survivor’s guilt may play a role [41, 42]. Moreover, the unaffected relatives consistently reported lower cancer worry than individuals with cancer. Given that cancer worry can motivate screening behavior [5], individuals with cancer may be more likely to undergo surveillance than those without cancer. Targeted educational interventions that emphasize the importance of surveillance are warranted for unaffected relatives carrying GPVs.

### Limitations

This study has some limitations. First, participants may have self-selected based on their interest in receiving MGPT results, as they all expressed a desire to know their outcomes, introducing the possibility of selection bias. Second, most probands carried *BRCA1/2* variants, and only one relative had a GPV in a gene distinct from that of the proband; the low prevalence of additional GPVs might have limited the scope of psychological impact assessment by MGPT. Third, the participants underwent genetic testing within the research framework. Some individuals in the cancer group had already undergone genetic testing or genetic counseling in clinical practice prior to study enrollment, and some had GPVs detected before participating in the study. Therefore, this study population represented a partially selected cohort, and the psychological responses might differ from those observed in routine clinical practice. Therefore, caution should be exercised when generalizing these findings. Fourth, the ePRO response rates for unaffected relatives and individuals with cancer were 62% and 52%, respectively, which raises the possibility of a response bias (Supplementary Table 4). Fifth, the majority of genetic tests for hereditary cancers are not covered by public health insurance. However, in the present study, genetic testing was provided free of charge through research funding, thereby eliminating financial concerns. These results may differ if the testing is offered as a clinical service.

## Conclusion

To our knowledge, this is the first study to evaluate the psychological impact of cascade testing using MGPT in unaffected first-degree relatives of individuals with hereditary cancer. Overall, MGPT was well tolerated: only 6.5% of relatives met criteria for clinically significant distress two weeks after result disclosure. Preexisting cancer worry was associated with higher post-disclosure distress, whereas the test result itself was not associated with high distress. Cancer worry did not change significantly following testing, and 41% of relatives exhibited persistent worry. These findings suggest that, while MGPT generally imposes minimal short-term psychological burden on unaffected relatives, routine psychological assessment beginning in the pre-test phase and tailored psychosocial support for high-risk subgroups remain important. Future studies should examine psychological outcomes among carriers of GPVs beyond *BRCA1/2* and among relatives found to carry GPVs not identified in the proband, to clarify their distinct support needs and inform targeted interventions.

## Supplementary information

Summary of Supplementary Information

Supplementary Table 1

Supplementary Table 2

Supplementary Table 3

Supplementary Table 4

Supplementary Table 5

Supplementary Table 6

Supplementary Table 7

Supplementary Table 8

Supplementary Figure 1

Supplementary Figure 2

COI disclosure_TK

COI disclosure_MW

COI disclosure_MY

COI disclosure_TY

COI disclosure_YN
