# Supplementary material for: Psychological distress and cancer worry in unaffected relatives undergoing cascade testing with multigene panel testing
Source: J Hum Genet. 2026 Mar 2;71(7):435–42. doi: 10.1038/s10038-026-01464-z (PMC13303072; doi:10.1038/s10038-026-01464-z)
Supplement: Supplementary file 1 — Summary of Supplementary Information [file 10038_2026_1464_MOESM1_ESM.docx]

**Summary of Supplementary Information**

**Supplementary Figure 1 BRANCH Study : overall framework** (.pdf)

The BRANCH study consisted of four cohorts. Peripheral blood was collected after informed consent and analyzed by FALCO Biosystems (Kyoto, Japan). Results were disclosed by attending physicians. Demographic and psychological data were collected at T0 (10 days after blood collection) and T1 (two weeks after result disclosure).

**Supplementary Figure 2 Participants in this sub-study** (.pdf)

Unaffected relatives were derived from Cohort C and individuals with cancer were derived from a subset of Cohort C and from Cohort D of the BRANCH study.

**Supplementary Table 1 Data collection** (.docx)

Demographic, clinical, and psychological data were collected using an electronic patient-reported outcome and electronic data-capture system before and after the disclosure of genetic testing results.

**Supplementary Table 2 List of genes** (.docx)

a. List of pre-defined genes

In this sub-study, we defined unaffected relatives as cancer-unaffected first-degree relatives of cancer-affected proband in whom GPVs were identified in pre-specified genes.

b. List of 35 genes included in the multigene panel testing

This MGPT includes hereditary tumor predisposition genes associated with increased risks of breast, ovarian, colorectal, and pancreatic cancers.

**Supplementary Table 3 Cancer worry scale and impact of events scale-revised** (.docx)

The table presents items assessing cancer worry (CWS) and genetic testing-related distress (IES-R). Unaffected relatives completed the Japanese version of the CWS (CWS-J) before and two weeks after result disclosure, and the IES-R two weeks after disclosure to evaluate psychological responses.

**Supplementary Table 4 Background of respondents and non-respondents** (.docx)

Background characteristics of respondents and non-respondents, stratified by unaffected relatives and individuals with cancer.

**Supplementary Table 5 Respondent characteristics** (.docx)

Characteristics of responders stratified by unaffected relatives (N = 123) and individuals with cancer (N = 67).

**Supplementary Table 6 Association between genetic testing-related distress and background factors** (.docx)

Association between background factors (demographic characteristics, clinical variables, and cancer worry) and genetic testing-related distress in the unaffected relatives. Statistical significance was set at *p* < 0.05.

**Supplementary Table 7 Cancer-unaffected first-degree relatives of individuals with hereditary cancer distribution stratified by cancer worry and genetic testing-related distress** (.docx)

Unaffected relatives were grouped into three categories based on post-disclosure CWS-J and IES-R scores. The table presents the distribution of clinical and sociodemographic characteristics. Cancer worry (CWS-J ≥14) and genetic testing-related distress (IES-R ≥25) were each classified as high or low using established cutoffs.

**Supplementary Table 8 Changes in cancer worry before and after disclosure of genetic testing results provided by BRANCH study** (.docx)

CWS-J before and after genetic test result disclosure are shown separately for unaffected relatives and individuals with cancer. The p-values from single-group t-tests assess whether score changes significantly differ from zero. Statistical significance was defined as p < 0.05.
