# Supplementary material for: Psychological distress and cancer worry in unaffected relatives undergoing cascade testing with multigene panel testing
Source: J Hum Genet. 2026 Mar 2;71(7):435–42. doi: 10.1038/s10038-026-01464-z (PMC13303072; doi:10.1038/s10038-026-01464-z)
Supplement: Supplementary file 2 — Supplementary Table 1 [file 10038_2026_1464_MOESM2_ESM.docx]

**Supplementary Table 1** Data collection and collection times

|  | **Baseline: 10 days after genetic testing (T0)** | 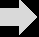 | **2 weeks after the results disclosure (T1)** |
| --- | --- | --- | --- |
| **Clinical and demographic variables (EDC)** | Gender |  | Genetic testing results provided by BRANCH study |
|  | Age |  |  |
|  | Cancer status^1^ |  |  |
|  | Recurrence and metastasis status^1^ |  |  |
|  | Previous *BRCA1/2* testing. |  |  |
|  | Ethnicity |  |  |
|  | Cancer of proband^2^ |  |  |
|  | Relationship with proband^2^ |  |  |
| **Demographic variables**  **(ePRO)** | Family history of cancer |  | Frequency of cancer risk discussions with family members |
|  | Education |  | Satisfaction with undergoing MGPT in BRANCH study |
|  | Having children |  |  |
|  | Marital status |  |  |
|  | Income |  |  |
|  | Cancer screening status |  |  |
|  | Cancer insurance^2^ |  |  |
|  | Intention to communicate genetic test results with family members |  |  |
| **Psychological variables (ePRO)** | Cancer worry (CWS-J) |  | CWS-J |
|  |  |  | Distress (IES-R)^2^ |

^1^Data were collected exclusively from individuals with cancer. ^2^Data were collected exclusively from cancer-unaffected first-degree relatives of individuals with hereditary cancer.

EDC, electronic data-capture system; ePRO, electronic patient-reported outcome; CWS-J, Japanese version of the Cancer Worry Scale; MGPT, multigene panel testing; IES-R, Impact of Event Scale-Revised.
