# Supplementary material for: Psychological distress and cancer worry in unaffected relatives undergoing cascade testing with multigene panel testing
Source: J Hum Genet. 2026 Mar 2;71(7):435–42. doi: 10.1038/s10038-026-01464-z (PMC13303072; doi:10.1038/s10038-026-01464-z)
Supplement: Supplementary file 3 — Supplementary Table 2 [file 10038_2026_1464_MOESM3_ESM.docx]

| **Supplementary Table 2** List of genes | | | |  | |  |  | |  | |  |
| --- | --- | --- | --- | --- | --- | --- | --- | --- | --- | --- | --- |
| a. List of pre-defined genes | | | |  | |  |  | |  | |  |
| *APC* | *ATM* | | *BAP1* | | *BARD1* | *BMPR1A* | | *BRCA1* | | *BRCA2* | |
| *BRIP1* | *CDH1* | | *CDK4* | | *CDKN2A* | *CHEK2* | | *DICER1* | | *EPCAM* | |
| *FH* | *FLCN* | | *MAX* | | *MEN1* | *MET* | | *MLH1* | | *MSH2* | |
| *MSH6* | *MUTYH ^1^* | | *NF1* | | *NF2* | *NTHL1* ^1^ | | *PALB2* | | *PMS2* | |
| *POLD1* | *POLE* | | *POT1* | | *PTCH1* | *PTEN* | | *RAD51C* | | *RAD51D* | |
| *RB1* | *RET* | | *SDHA* | | *SDHAF2* | *SDHB* | | *SDHC* | | *SDHD* | |
| *SMAD4* | *SMARCA4* | | *SMARCB1* | | *STK11* | *SUFU* | | *TMEM127* | | *TP53* | |
| *TSC1* | *TSC2* | | *VHL* | | *WT1* |  | |  | |  | |
| ^1^Individuals with only a homozygote or compound heterozygote pathogenic variants of *MUTYH* and *NTHL1* were classified as having hereditary cancer for this study. | | | | | | | | | | | |
|  | |  | |  | |  |  | |  | |  |
| b. List of 35 genes included in the multigene panel testing | | | | | |  |  | |  | |  |
| *APC* | *ATM* | | *AXIN2* | | *BARD1* | *BMPR1A* | | *BRCA1* | | *BRCA2* | |
| *BRIP1* | *CDH1* | | *CDKN2A* | | *CDK4* | *CHEK2* | | *EPCAM* | | *GALNT12* | |
| *GREM1* | *HOXB13* | | *MLH1* | | *MSH2* | *MSH3* ^1^ | | *MSH6* | | *MUTYH ^1^* | |
| *NBN* | *NTHL1* ^1^ | | *PALB2* | | *POLD1* | *POLE* | | *PMS2* | | *PTEN* | |
| *RAD51C* | *RAD51D* | | *RNF43* | | *RPS20* | *SMAD4* | | *STK11* | | *TP53* | |
| ^1^Individuals with only heterozygote pathogenic variants of *MSH3, MUTYH,* and *NTHL1* were considered as negative for this analysis. | | | | | | | | | | | |
