# Supplementary material for: Psychological distress and cancer worry in unaffected relatives undergoing cascade testing with multigene panel testing
Source: J Hum Genet. 2026 Mar 2;71(7):435–42. doi: 10.1038/s10038-026-01464-z (PMC13303072; doi:10.1038/s10038-026-01464-z)
Supplement: Supplementary file 4 — Supplementary Table 3 [file 10038_2026_1464_MOESM4_ESM.docx]

| **Supplementary Table 3** Cancer worry scale and impact of events scale-revised |
| --- |
|  |
| **Cancer worry scale (CWS)** |
| Answer for the last 7 days |
| 1. How often have you thought about your chances of getting cancer(again)? |
| 2. Have these thoughts affected your mood? |
| 3. Have these thoughts interfered with your ability to do daily activities? |
| 4. How concerned are you about the possibility of getting cancer one day? |
| 5. How often do you worry about developing cancer? |
| 6. How much of a problem is this worry? |
| 7. How often do you worry about the chance of family members developing cancer? |
| 8. How concerned are you about the possibility that you will ever need surgery (again)? |
|  |
| **Impact of events scale-revised (IES-R)** |
| Answer for the last 7 days respect to event* |
| *Event is the "disclosure of genetic testing results in the BRANCH study." |
| 1. Any reminder brought back feelings about it |
| 2. I had trouble staying asleep |
| 3. Other things kept making me think about it. |
| 4. I felt irritable and angry |
| 5. I avoided letting myself get upset when I thought about it or was reminded of it |
| 6. I thought about it when I didn’t mean to |
| 7. I felt as if it had not happened or was not real. |
| 8. I stayed away from reminders of it. |
| 9. Pictures about it popped into my mind. |
| 10. I was jumpy and easily startled. |
| 11. I tried not to think about it. |
| 12. I was aware that I still had a lot of feelings about it, but I didn't deal with them. |
| 13. My feelings about it were kind of numb. |
| 14. I found myself acting or feeling like I was back at that time. |
| 15. I had trouble falling asleep. |
| 16. I had waves of strong feelings about it. |
| 17. I tried to remove it from my memory. |
| 18. I had trouble concentrating. |
| 19. Reminders of it caused me to have physical reactions, such as sweating, trouble breathing, nausea, or a pounding heart. |
| 20. I had dreams about it. |
| 21. I felt watchful and on guard. |
| 22. I tried not to talk about it. |
