# Supplementary material for: Psychological distress and cancer worry in unaffected relatives undergoing cascade testing with multigene panel testing
Source: J Hum Genet. 2026 Mar 2;71(7):435–42. doi: 10.1038/s10038-026-01464-z (PMC13303072; doi:10.1038/s10038-026-01464-z)
Supplement: Supplementary file 5 — Supplementary Table 4 [file 10038_2026_1464_MOESM5_ESM.docx]

| **Supplementary Table 4** Background of respondents and non-respondents | | | | | | |
| --- | --- | --- | --- | --- | --- | --- |
|  | Unaffected relatives (N = 198) | | | Individuals with cancer (N = 128) | | |
|  | Non-respondents | Respondents | *p*-value | Non-respondents | Respondents | *p*-value |
|  | n = 75 (%) | n = 123 (%) |  | n = 61 (%) | n = 67 (%) |  |
| **Age** |  |  |  |  |  |  |
| Median (range) | 35 (18-77) | 44 (18-76) | 0.0127 | 51 (25-75) | 50 (20-77) | 0.4130 |
| **Gender** |  |  |  |  |  |  |
| Male | 34 (45.3) | 42 (34.1) | 0.1164 | 6 (9.8) | 2 (3.0) | 0.1098 |
| Female | 41 (54.7) | 81 (65.9) |  | 55 (90.2) | 65 (97.0) |  |
| **Cancer diagnosis** |  |  |  |  |  |  |
| No | 75 (100) | 123 (100) | - | 0 (0) | 0 (0) | - |
| Yes | 0 (0) | 0 (0) |  | 61 (100) | 67 (100) |  |
| **Cancer status ^1^** |  |  |  |  |  |  |
| Breast | - | - |  | 43 (70.5) | 54 (80.6) |  |
| Pancreas | - | - |  | 6 (9.8) | 4 (6.0) |  |
| Prostate | - | - |  | 1 (1.6) | 0 (0) |  |
| Ovary | - | - |  | 9 (14.8) | 9 (13.4) |  |
| Others | - | - |  | 2 (3.3) | 0 (0) |  |
| **Genetic testing results provided by BRANCH study** | | | | | | |
| Negative | 36 (48.0) | 44 (35.8) | 0.0441 | 14 (23.0) | 23 (34.3) | 0.0167 |
| GPV | 33 (44.0) | 54 (43.9) |  | 42 (68.8) | 30 (44.8) |  |
| VUS | 6 (8.0) | 25 (20.3) |  | 5 (8.2) | 14 (20.9) |  |
| **Relationship with proband ^2^** |  |  |  |  |  |  |
| Father | 2 (2.7) | 4 (3.3) | 0.4060 | - | - | - |
| Mother | 4 (5.3) | 5 (4.1) |  | - | - |  |
| Children | 50 (66.7) | 69 (56.1) |  | - | - |  |
| Sibling /brother | 19 (25.3) | 45 (36.6) |  | - | - |  |
| **Recurrence ^1^** |  |  |  |  |  |  |
| No | - | - | - | 50 (82.0) | 58 (86.6) | 0.4741 |
| Yes | - | - |  | 11 (18.0) | 9 (13.4) |  |
| **Metastasis ^1^** |  |  |  |  |  |  |
| No | - | - | - | 42 (68.9) | 51 (76.1) | 0.6370 |
| Yes | - | - |  | 16 (26.2) | 13 (19.4) |  |
| Unknown | - | - |  | 3 (4.9) | 3 (4.5) |  |
| **Genetic counseling** |  |  |  |  |  |  |
| No | 3 (4.0) | 3 (2.4) | 0.5342 | 6 (9.8) | 0 (0) | 0.0086 |
| Yes | 72 (96.0) | 120 (97.6) |  | 55 (90.2) | 67 (100) |  |
| **Previous genetic testing** |  |  |  |  |  |  |
| No | 75 (100) | 123 (100) | - | 3 (4.9) | 2 (3.0) | 0.5729 |
| Yes | 0 (0) | 0 (0) |  | 58 (95.1) | 65 (97.0) |  |
| **Previous *BRCA1, BRCA2* genetic testing results** | | | | | | |
| Negative | - | - |  | 23 (37.7) | 37 (55.2) | 0.0488 |
| GPV | - | - | - | 35 (57.4) | 25 (37.3) |  |
| VUS | - | - |  | 0 (0) | 3 (4.5) |  |
| Unaffected relatives, cancer-unaffected first-degree relatives of individuals with hereditary cancer; GPV, Germline pathogenic variant; VUS, Variant of uncertain significance | | | | | | |
| ^1^ Data were collected exclusively from individuals with cancer; ^2^ Data were collected exclusively from unaffected relatives | | | | | | |
