# Supplementary material for: Psychological distress and cancer worry in unaffected relatives undergoing cascade testing with multigene panel testing
Source: J Hum Genet. 2026 Mar 2;71(7):435–42. doi: 10.1038/s10038-026-01464-z (PMC13303072; doi:10.1038/s10038-026-01464-z)
Supplement: Supplementary file 6 — Supplementary Table 5 [file 10038_2026_1464_MOESM6_ESM.docx]

**Supplementary Table 5** Respondent characteristics

|  | Unaffected relatives | Individuals with cancer | *p*-value |
| --- | --- | --- | --- |
|  | n = 123 (%) | n = 67 (%) |  |
| **Age** |  |  |  |
| Median (range) | 44 (18-76) | 50 (20-77) | 0.0011 |
| **Gender** |  |  |  |
| Male | 42 (34.2) | 2 (3.0) | <0.0001 |
| Female | 81 (65.9) | 65 (97.0) |  |
| **Cancer diagnosis** |  |  |  |
| No | 123 (100) | 0 (0) | - |
| Yes | 0 (0) | 67 (100) |  |
| **Cancer status^1^** |  |  |  |
| Breast | - | 54 (80.6) |  |
| Pancreas | - | 4 (6.0) |  |
| Ovary | - | 9 (13.4) |  |
| **Genetic testing results provided by BRANCH study** | | |  |
| Negative | 44 (35.8) | 23 (34.3) | 0.9802 |
| GPV | 54 (43.9) | 30 (44.8) |  |
| VUS | 25 (20.3) | 14 (20.9) |  |
| **Germline pathogenic variants^3^** |  | |  |
| *ATM* | 2 (3.7) | 0 (0) |  |
| *BRCA1* | 14 (25.9) | 14 (46.7) |  |
| *BRCA2* | 32 (59.3) | 13 (43.3) |  |
| *MLH1* | 1 (1.9) | 0 (0) |  |
| *MSH2* | 1 (1.9) | 1 (3.3) |  |
| *MSH6* | 1 (1.9) | 0 (0) |  |
| *PALB2* | 2 (3.7) | 3 (10.0) |  |
| *PMS2* | 1 (1.9) | 0 (0) |  |
| *RAD51D* | 1 (1.9) | 1 (3.3) |  |
| *SDHB* | 1 (1.9) | 0 (0) |  |
| *TP53* | 0 (0) | 1 (3.3) |  |
| **Marital Status (partner)** |  | |  |
| No | 48 (39.0) | 16 (23.9) | 0.0752 |
| Yes | 74 (60.2) | 51 (76.1) |  |
| No answer | 1 (0.8) | 0 (0) |  |
| **Children** |  |  |  |
| No | 75 (61.0) | 48 (71.6) | 0.1415 |
| Yes | 48 (39.0) | 19 (28.4) |  |
| **Education level** |  |  |  |
| ～Junior colleges | 59 (48.0) | 48 (71.6) | 0.0051 |
| University～ | 63 (51.2) | 18 (26.9) |  |
| No answer | 1 (0.8) | 1 (1.5) |  |
| **Income** |  |  |  |
| ~Five million | 47 (38.2) | 21 (31.3) | 0.3905 |
| Five million~ | 57 (46.3) | 38 (56.7) |  |
| No answer | 19 (15.5) | 8 (11.9) |  |
| **Frequency of cancer risk discussion with family members** | | |  |
| A lot | 24 (19.5) | 13 (19.4) | 0.6135 |
| Somewhat | 68 (55.3) | 39 (58.2) |  |
| A little | 24 (19.5) | 9 (13.4) |  |
| Not at all | 6 (4.9) | 6 (9.0) |  |
| Don't remember | 1 (0.8) | 0 (0) |  |
| **Ethnicity** |  |  |  |
| East Asian | 123 (100) | 67 (100) | - |
| **Genetic counseling** |  | |  |
| No | 3 (2.4) | 0 (0) | 0.1976 |
| Yes | 120 (97.6) | 67 (100) |  |
| **Previous genetic testing** |  | |  |
| No | 123 (100) | 2 (3.0) | <0.0001 |
| Yes | 0 (0) | 65 (97.0) |  |
| **Previous *BRCA1* and *BRCA2* genetic testing results^4^** | | |  |
| Negative | **-** | 40 (59.7) |  |
| GPV | - | 25 (37.3) |  |
| **Relationship with proband^2^** |  | |  |
| Father | 4 (3.3) | - |  |
| Mother | 5 (4.1) | - |  |
| Children | 69 (56.1) | - |  |
| Sibling /brother | 45 (36.6) | - |  |
| **Cancer of proband^2^** |  | |  |
| Breast | 39 (31.7) | - |  |
| Pancreas | 27 (22.0) | - |  |
| Ovary | 25 (20.3) | - |  |
| Prostate | 4 (3.3) | - |  |
| Others | 28 (22.8) | - |  |
| **Recurrence^1^** |  |  |  |
| No | - | 58 (86.6) |  |
| Yes | - | 9 (13.4) |  |
| **Metastasis^1^** |  |  |  |
| No | - | 51 (76.1) |  |
| Yes | - | 13 (19.4) |  |
| Unknown | - | 3 (4.5) |  |
| **Family history in second degree** |  | |  |
| No | 0 | 4 (6.0) |  |
| Yes | 123 (100) | 63 (94.0) |  |
| **Satisfaction with multigene panel testing in BRANCH study** | | |  |
| Satisficed | 91 (74.0) | 43 (64.2) | 0.0081 |
| Somewhat satisfied | 14 (11.4) | 19 (28.4) |  |
| Neither. | 18 (14.6) | 5 (7.5) |  |
| Somewhat unsatisfied | 0 (0) | 0 (0) |  |
| Unsatisfied | 0 (0) | 0 (0) |  |

Unaffected relatives, cancer-unaffected first-degree relatives of individuals with hereditary cancer; GPV, Germline pathogenic variant; VUS, Variant of uncertain significance

^1^Data were collected exclusively from individuals with cancer.

^2^Data were collected exclusively from the unaffected relatives.

^3^Two unaffected relatives and three individuals with cancer carried GPVs in two distinct genes.

^4^n = 65 (previous genetic testing was "Yes")
