# Supplementary material for: Psychological distress and cancer worry in unaffected relatives undergoing cascade testing with multigene panel testing
Source: J Hum Genet. 2026 Mar 2;71(7):435–42. doi: 10.1038/s10038-026-01464-z (PMC13303072; doi:10.1038/s10038-026-01464-z)
Supplement: Supplementary file 7 — Supplementary Table 6 [file 10038_2026_1464_MOESM7_ESM.docx]

| **Supplementary Table 6** Association between genetic testing-related distress and background factors | | | | |
| --- | --- | --- | --- | --- |
|  | n | Mean　(SD) | *p*-value^*1^ | *p*-value^*2^ |
|  |  |  | Univariable | Multivariable |
| **Age** |  |  |  |  |
| <40 | 51 | 5.9 (9.4) | 0.9795 | 0.4053 |
| ≥40 | 72 | 5.8 (9.0) |  |  |
| **Gender** |  |  |  |  |
| Male | 42 | 5.3 (9.2) | 0.617 | 0.8191 |
| Female | 81 | 6.1 (9.2) |  |  |
| **Relationship with proband** |  |  |  |  |
| Parents | 9 | 2.0 (1.9) | 0.0517 | 0.0552 |
| Children | 69 | 7.6 (11.1) |  |  |
| Sibling/brother | 45 | 4.0 (5.4) |  |  |
| **Genetic testing results provided by BRANCH study** |  |  |  |  |
| Negative | 44 | 4.8 (6.4) | 0.0205 | 0.1386 |
| GPV | 54 | 8.2 (11.6) |  |  |
| VUS | 25 | 2.4 (5.4) |  |  |
| **Children** |  |  |  |  |
| No | 75 | 6.6 (10.4) | 0.2660 | 0.3564 |
| Yes | 48 | 4.7 (6.5) |  |  |
| **Marital Status (partner)** |  |  |  |  |
| No | 48 | 5.5 (10.1) | 0.6862 | 0.3059 |
| Yes | 74 | 6.1 (8.5) |  |  |
| **Education level** |  |  |  |  |
| ～Junior colleges | 59 | 5.0 (7.0) | 0.3275 | 0.9150 |
| University～ | 63 | 6.7 (10.8) |  |  |
| **Income (yen)** |  |  |  |  |
| Five million | 47 | 3.9 (4.8) | 0.1576 | 0.1099 |
| Five million~ | 57 | 7.3 (11.6) |  |  |
| No answer | 19 | 6.5 (8.5) |  |  |
| **Cancer type of the proband** |  |  |  |  |
| Breast | 39 | 5.2 (10.3) | 0.4108 | 0.3748 |
| Pancreas | 27 | 8.7 (10.1) |  |  |
| Ovary | 25 | 5.5 (10.1) |  |  |
| Prostate | 4 | 1.5 (0.6) |  |  |
| Others | 28 | 4.9 (5.3) |  |  |
| **Cancer screening status** |  |  |  |  |
| No | 66 | 5.2 (8.2) | 0.4394 | 0.5614 |
| Yes | 57 | 6.5 (10.1) |  |  |
| **Insurance** |  |  |  |  |
| No | 77 | 6.2 (9.6) | 0.7920 | 0.6269 |
| Yes | 33 | 5.6 (9.7) |  |  |
| No answer | 13 | 4.4 (4.3) |  |  |
| **Frequency of cancer risk discussion with family members** | |  |  |  |
| A lot/Somewhat | 92 | 6.9 (9.9) | 0.0211 | 0.3647 |
| A little/Not at all/Don't remember | 31 | 2.6 (5.5) |  |  |
| **Intention to communicate about undergoing genetic testing with family members** | | | | |
| Yes/Already | 118 | 5.6 (8.1) | 0.1013 | 0.1803 |
| No/Unknown | 5 | 12.4 (24.0) |  |  |
| **Family history in second degree^1^** |  |  |  |  |
| No | 0 | - | - | - |
| Yes | 123 | 5.8 (9.1) |  |  |
| **CWS-J score *^3^ (T0)^*4^** |  |  |  |  |
| Low | 54 | 1.8 (2.7) | <0.0001 | 0.0003 |
| High | 69 | 9.0 (11.0) |  |  |
| GPV, Germline pathogenic variant; VUS, Variant of uncertain significance; CWS-J, Japanese version of the Cancer Worry Scale | | | | |
| *1 t-test or analysis of variance (ANOVA) |  |  |  |  |
| *2 Multiple regression analysis (121 participants for whom complete covariate data were available.) | | | | |
| *3 Low: CWS-J score is 13 or less, High: CWS-J score is 14 or over | |  |  |  |
| *4 T0: baseline |  |  |  |  |
| ^1^ "Family history in second degree" was not included in the multivariable analysis | | |  |  |
