# Supplementary material for: Psychological distress and cancer worry in unaffected relatives undergoing cascade testing with multigene panel testing
Source: J Hum Genet. 2026 Mar 2;71(7):435–42. doi: 10.1038/s10038-026-01464-z (PMC13303072; doi:10.1038/s10038-026-01464-z)
Supplement: Supplementary file 8 — Supplementary Table 7 [file 10038_2026_1464_MOESM8_ESM.docx]

| **Supplementary Table 7** Cancer-unaffected first-degree relatives of individuals with hereditary cancer distribution stratified by cancer worry and genetic testing related-distress | | | | |
| --- | --- | --- | --- | --- |
| CWS-J/IES-R | High/High | High/Low | Low/Low |  |
|  | n (%) | n (%) | n (%) | N |
|  | 8 | 61 | 54 | 123 |
| Age |  |  |  |  |
| Median (range) | 34(21-47) | 45(22-76) | 44(18-74) | 123 |
| Gender |  |  |  |  |
| Male | 3(7.1) | 20(47.6) | 19(45.2) | 42 |
| Female | 5(6.2) | 41(50.6) | 35(43.2) | 81 |
| Genetic testing results provided by BRANCH study | | | | |
| Negative | 2(4.5) | 21(47.7) | 21(47.7) | 44 |
| GPV | 6(11.1) | 28(51.9) | 20(37.0) | 54 |
| VUS | 0(0) | 12(48.0) | 13(52.0) | 25 |
| Marital Status (partner) |  |  |  |  |
| Yes | 4(5.4) | 41(55.4) | 29(39.2) | 74 |
| No | 4(8.3) | 19(39.6) | 25(52.1) | 48 |
| No answer | 0 | 1(100) | 0 | 1 |
| Children |  |  |  |  |
| Yes | 2(4.2) | 22(45.8) | 24(50.0) | 48 |
| No | 6(8.1) | 39(52.7) | 29(39.2) | 74 |
| Relationship with proband |  |  |  |  |
| Parents | 0 | 4(44.4) | 5(56.6) | 9 |
| Children | 8(11.6) | 31(44.9) | 30(43.5) | 69 |
| Sibling/brother | 0 | 26(57.8) | 19(42.2) | 45 |
| Frequency of cancer risk discussion with family members |  |  |  |  |
| A lot/Somewhat | 7(7.6) | 54(58.7) | 31(33.7) | 92 |
| A little/Not at all/Don't remember | 1(3.2) | 7(22.6) | 23(74.2) | 31 |
| Backgrounds of proband |  |  |  |  |
| Cancer of proband |  |  |  |  |
| Breast | 2(5.1) | 12(30.8) | 25(64.1) | 39 |
| Pancreas | 4(14.8) | 14(51.9) | 9(33.3) | 27 |
| Ovary | 2(8.0) | 13(52.0) | 10(40.0) | 25 |
| Prostate | 0(0) | 2(50.0) | 2(50.0) | 4 |
| Stage of proband’s cancer |  |  |  |  |
| Ⅳ | 2(4.9) | 25(61.0) | 14 (34.1) | 41 |
| ~Ⅲ | 6(8.1) | 32(43.2) | 36(48.6) | 74 |
| Unknown | 0(0) | 4(50.0) | 4(50.0) | 8 |
| GPV of proband |  |  |  |  |
| *BRCA* or *BRCA2* | 6(6.2) | 45(46.4) | 46(47.4) | 97 |
| Others | 2(7.7) | 16(61.5) | 8(30.8) | 26 |

GPV, Germline pathogenic variant; VUS, Variant of uncertain significance.
