# Supplementary material for: Psychological distress and cancer worry in unaffected relatives undergoing cascade testing with multigene panel testing
Source: J Hum Genet. 2026 Mar 2;71(7):435–42. doi: 10.1038/s10038-026-01464-z (PMC13303072; doi:10.1038/s10038-026-01464-z)
Supplement: Supplementary file 9 — Supplementary Table 8 [file 10038_2026_1464_MOESM9_ESM.docx]

| **Supplementary Table 8** Changes in cancer worry before and after disclosure of genetic testing results provided by BRANCH study |
| --- |

|  | Unaffected relatives | | | | Individuals with cancer | | | |
| --- | --- | --- | --- | --- | --- | --- | --- | --- |
|  | T0 | T1 | Change in CWS-J  (SD) | *p*-value^1^ | T0 | T1 | Change in CWS-J  (SD) | *p*-value^1^ |
| All | 14.1 | 14.6 | 0.4(4.2) | 0.2728 | 18.8 | 18.2 | -0.6(4.0) | 0.1981 |
| Negative | 13.9 | 14.1 | 0.2(3.5) | 0.6731 | 19 | 18.6 | -0.4(3.6) | 0.5646 |
| GPV | 14 | 14.9 | 0.9(4.9) | 0.1873 | 18.9 | 18.1 | -0.8(4.1) | 0.3132 |
| VUS | 14.8 | 14.5 | -0.3(3.5) | 0.6912 | 18.4 | 17.6 | -0.7(4.9) | 0.5939 |

| Unaffected relatives, cancer-unaffected first-degree relatives of individuals with hereditary cancer; GPV, Germline pathogenic variant; VUS, Variant of uncertain significance; CWS-J, Japanese version of the Cancer Worry Scale |
| --- |
| T0: baseline, T1: Two weeks after genetic testing results disclosure |
| ^1^Single group t-test on whether the change is zero or not |
