# Supplementary material for: Psychological distress and cancer worry in unaffected relatives undergoing cascade testing with multigene panel testing
Source: J Hum Genet. 2026 Mar 2;71(7):435–42. doi: 10.1038/s10038-026-01464-z (PMC13303072; doi:10.1038/s10038-026-01464-z)
Supplement: Supplementary file 10 — Supplementary Figure 1 [file 10038_2026_1464_MOESM10_ESM.pdf]

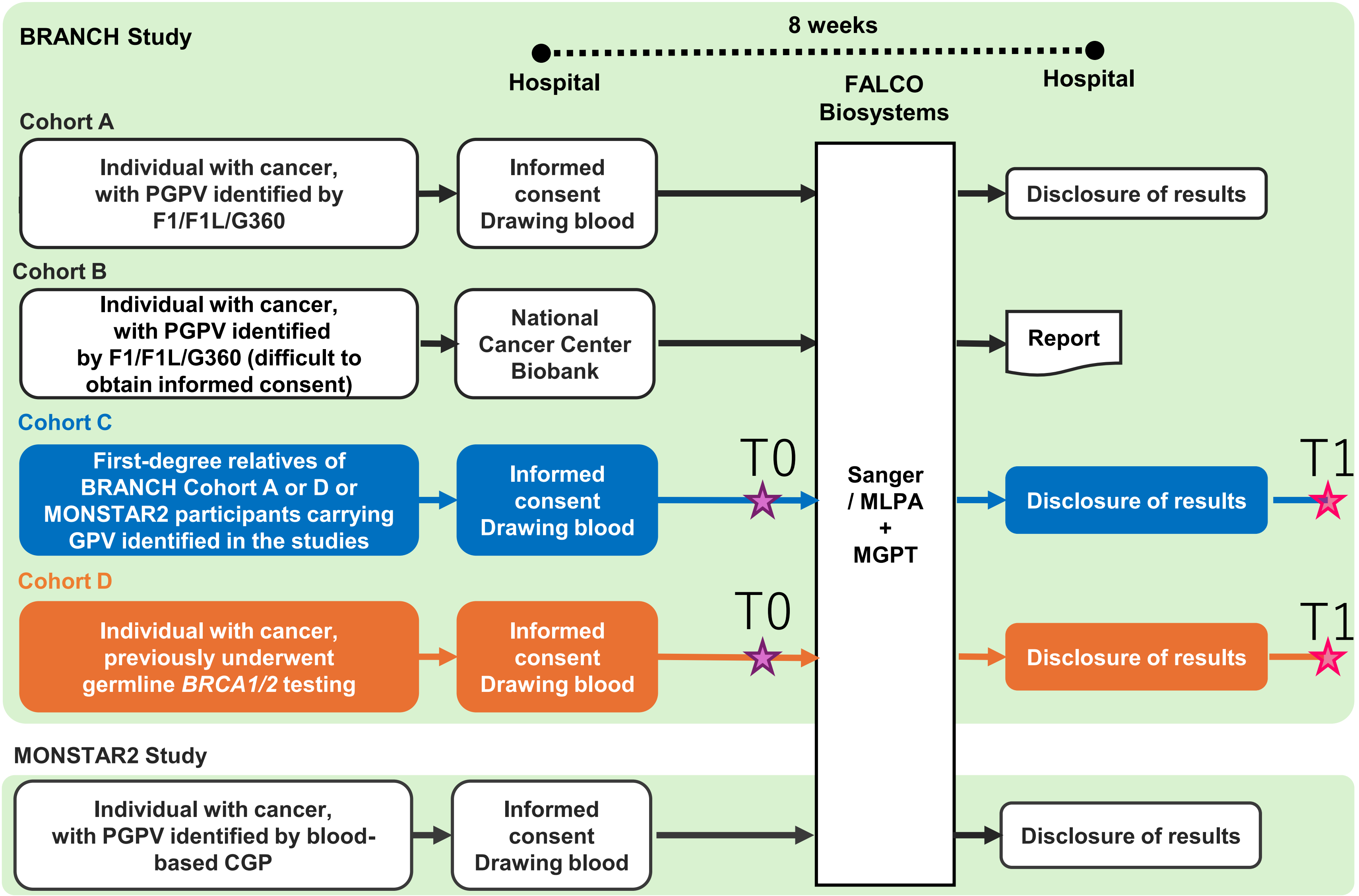

Purple Star, 10 days post-genetic testing (T0): [Data collection] Clinical and demographic characteristics, Psychological scales
Pink Star, 2 weeks after disclosure of results (T1): [Data collection] Demographic characteristics and psychological scales
PGPV, putative/presumed germline pathogenic variant; F1, Foundation one CDx; F1L, Foundation one CDx; G360, Gurdant 360; CGP, comprehensive genome profiling; MLPA, multiplex ligation-dependent probe amplification; MGPT, multigene panel testing; GPV, germline pathogenic variant

Supplementary figure 1 BRANCH Study : overall framework
