# Supplementary figures and images for: Psychological distress and cancer worry in unaffected relatives undergoing cascade testing with multigene panel testing
Source: J Hum Genet. 2026 Mar 2;71(7):435–42. doi: 10.1038/s10038-026-01464-z (PMC13303072; doi:10.1038/s10038-026-01464-z)

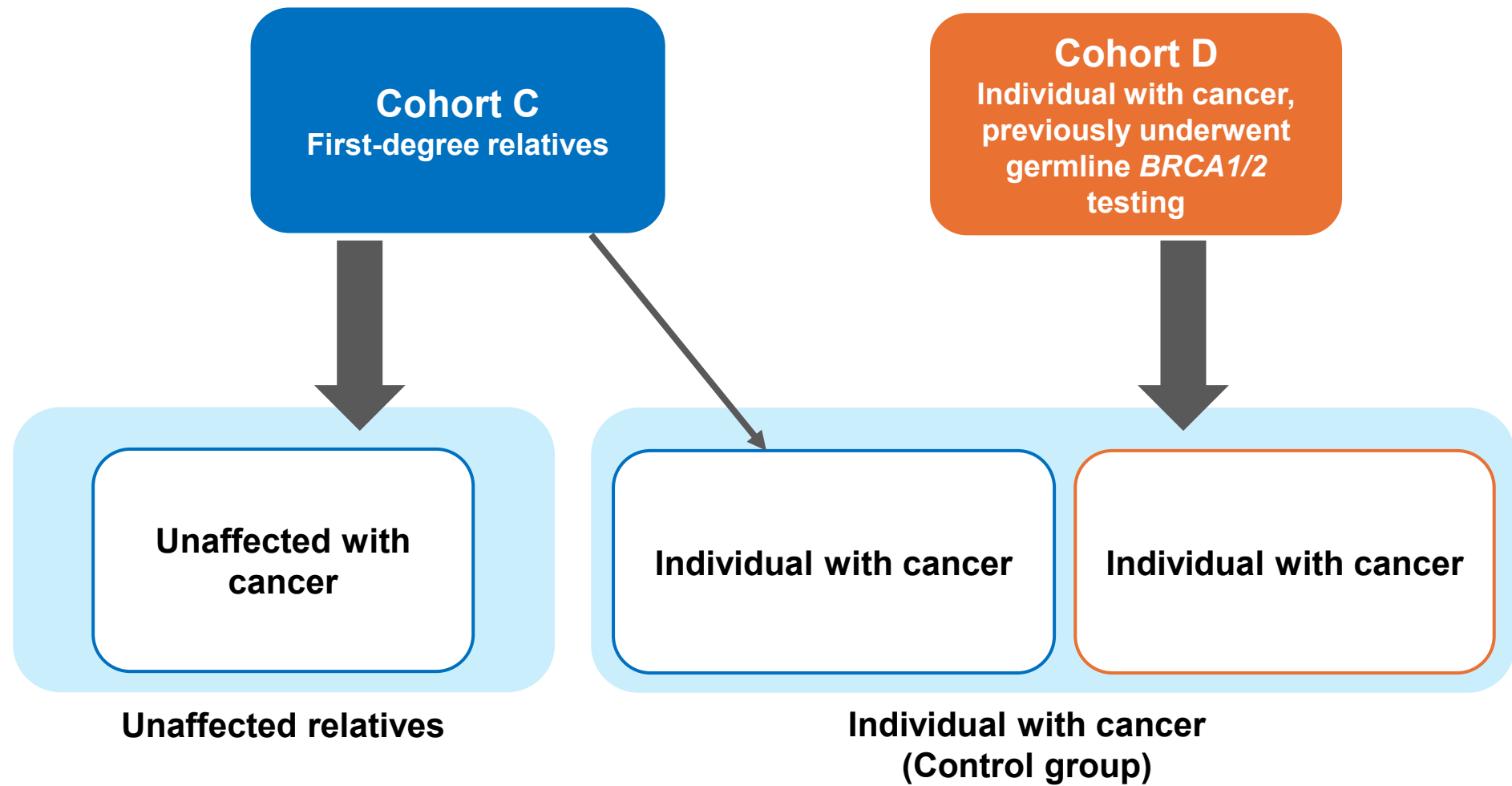

**Supplementary figure 2 Participants in this sub-study**

Supplement: Supplementary file 11 — Supplementary Figure 2 [file 10038_2026_1464_MOESM11_ESM.pdf]
